# Suppression of Cation Intermixing Highly Boosts the Performance of Core–Shell Lanthanide Upconversion Nanoparticles

**DOI:** 10.1021/jacs.3c03019

**Published:** 2023-08-07

**Authors:** Fuhua Huang, Niusha Bagheri, Li Wang, Hans Ågren, Jinglai Zhang, Rui Pu, Qiuqiang Zhan, Yuhan Jing, Wen Xu, Jerker Widengren, Haichun Liu

**Affiliations:** † Department of Applied Physics, KTH Royal Institute of Technology, S-10691 Stockholm, Sweden; ‡ College of Chemistry and Molecular Sciences, Henan University, Kaifeng, Henan 475004, P. R. China; § Henan Center for Outstanding Overseas Scientists, Henan University, Kaifeng 475004, P. R. China; ∥ Centre for Optical and Electromagnetic Research, Guangdong Provincial Key Laboratory of Optical Information Materials and Technology, South China Academy of Advanced Optoelectronics, South China Normal University, Guangzhou 510006, P.R. China; ⊥ MOE Key Laboratory of Laser Life Science, Guangdong Engineering Research Centre of Optoelectronic Intelligent Information Perception, South China Normal University, Guangzhou 510631, P.R. China; # Key Laboratory of New Energy and Rare Earth Resource Utilization of State Ethnic Affairs Commission, Key Laboratory of Photosensitive Materials & Devices of Liaoning Province, School of Physics and Materials Engineering, Dalian Minzu University, 18 Liaohe West Road, Dalian 11660, P.R. China

## Abstract

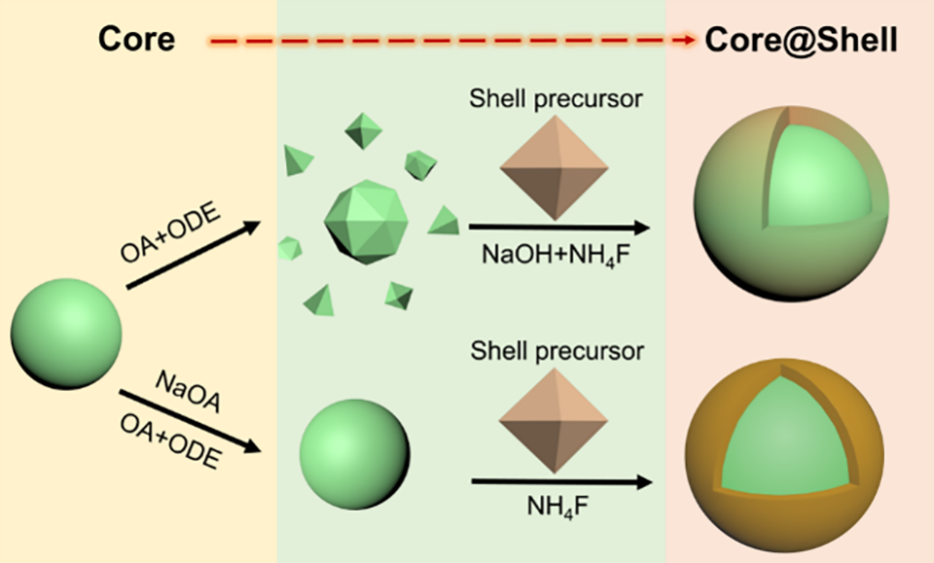

Lanthanide upconversion nanoparticles (UCNPs) have been extensively explored as biomarkers, energy transducers, and information carriers in wide-ranging applications in areas from healthcare and energy to information technology. In promoting the brightness and enriching the functionalities of UCNPs, core–shell structural engineering has been well-established as an important approach. Despite its importance, a strong limiting issue has been identified, namely, cation intermixing in the interfacial region of the synthesized core–shell nanoparticles. Currently, there still exists confusion regarding this destructive phenomenon and there is a lack of facile means to reach a delicate control of it. By means of a new set of experiments, we identify and provide in this work a comprehensive picture for the major physical mechanism of cation intermixing occurring in synthesis of core–shell UCNPs, i.e., partial or substantial core nanoparticle dissolution followed by epitaxial growth of the outer layer and ripening of the entire particle. Based on this picture, we provide an easy but effective approach to tackle this issue that enables us to produce UCNPs with highly boosted optical properties.

## Introduction

Lanthanide upconversion nanoparticles (UCNPs) have emerged as an important group of photoluminescent nanomaterials over the last twenty years.^1−4^ Their unique optical properties of converting low-energy near-infrared (NIR) photons into higher-energy NIR, visible or even ultraviolet ones have opened new avenues in wide-ranging applications, covering healthcare,^5−9^ information technology,^10^ and energy.^11,12^ In developing the optical properties of UCNPs, core–shell structural engineering has been well-established as a powerful strategy to boost the brightness and enrich the functionalities of the UCNPs.^13^ As an example, coating the surface of UCNPs with an inert shell of a few nanometers in thickness can readily protect them from surface quenching and so increase the upconversion luminescence (UCL) intensity by more than one order of magnitude.^14−17^ Construction of a core–(multi)shell structure can also regulate the complex interplay of lanthanide interactions^18^ and integrate functionalities of multiple elements into single nanoplatforms.^1,3,19^

Despite the importance of core–shell engineering in the development of innovative UCNPs, confusion still exists regarding the structural integrity and heterogeneity of the core–shell nanoparticles, much owing to the discrepancies between previously reported results. Limiting issues have definitely been identified with this strategy in the past years. The core–shell UCNPs have been most widely synthesized by the heating up method using pre-synthesized core particles as seeds for the growth of the shell,^20^ and (the notion of) an epitaxial growth of the shell onto the core (conceptually leading to sharp chemical and structural interfaces) has then been widely employed. On the one hand, many works have used the description of well-separated core–shells,^21−24^ the applicability of which is also partially supported by high-resolution transmission electron microscopy (TEM) characterization.^24^ On the other hand, it has also been observed that the (actual) “epitaxial”-growth of the shell may be oversimplified in certain circumstances and that it very often stands in disagreement with the corresponding published structural data. Strong evidence has been found that significant cation intermixing in the core–shell UCNPs can occur, which can lead to a much extended interfacial region, rather than a sharp interface between the core and the shell.^25−31^ Such cation intermixing can have a very deleterious impact on the optical performance of core–shell UCNPs. For instance, the diffusion of optically active ions into the shielding shell calls for the need of a considerable thickness of the inert layer to reach complete isolation of the active cores from environmental quenching channels.^32^ Although getting increased brightness, the application of such nanoparticles in biolabeling and imaging may be limited due to their larger size, where small size nanoparticles are generally desired. In addition, cation intermixing can also lead to severe luminescence quenching due to complex cross-relaxation interaction or other effects between lanthanide ions, e.g., the luminescence quenching of various lanthanide emitters caused by a small amount of Pr^3+^ ions.^33^ In our view, the upconversion community has not reached a full understanding of the possible elemental migration of lanthanide ions in core–shell nanoparticles and accordingly lacks a facile means to achieve satisfactory control of this phenomenon to produce well structurally defined and high-performance core–shell nanostructures.

In this work, we explored the stability of UCNPs in synthetic solvents at elevated temperatures, relevant to the nanoparticle synthesis conditions. We have identified seed core nanoparticle dissolution as the major mechanism for the cation intermixing in core–shell UCNPs and have obtained a comprehensive understanding of it. We report under which conditions it happens and to which parameters it is subject to. More importantly, we have established a facile route to get full control of this harmful process, which helps us obtain core–shell UCNPs with highly promoted performance.

## Results and Discussion

### Dissolution of Upconversion Nanoparticles in Synthetic Solvents at High Temperature

**Figure 1:**
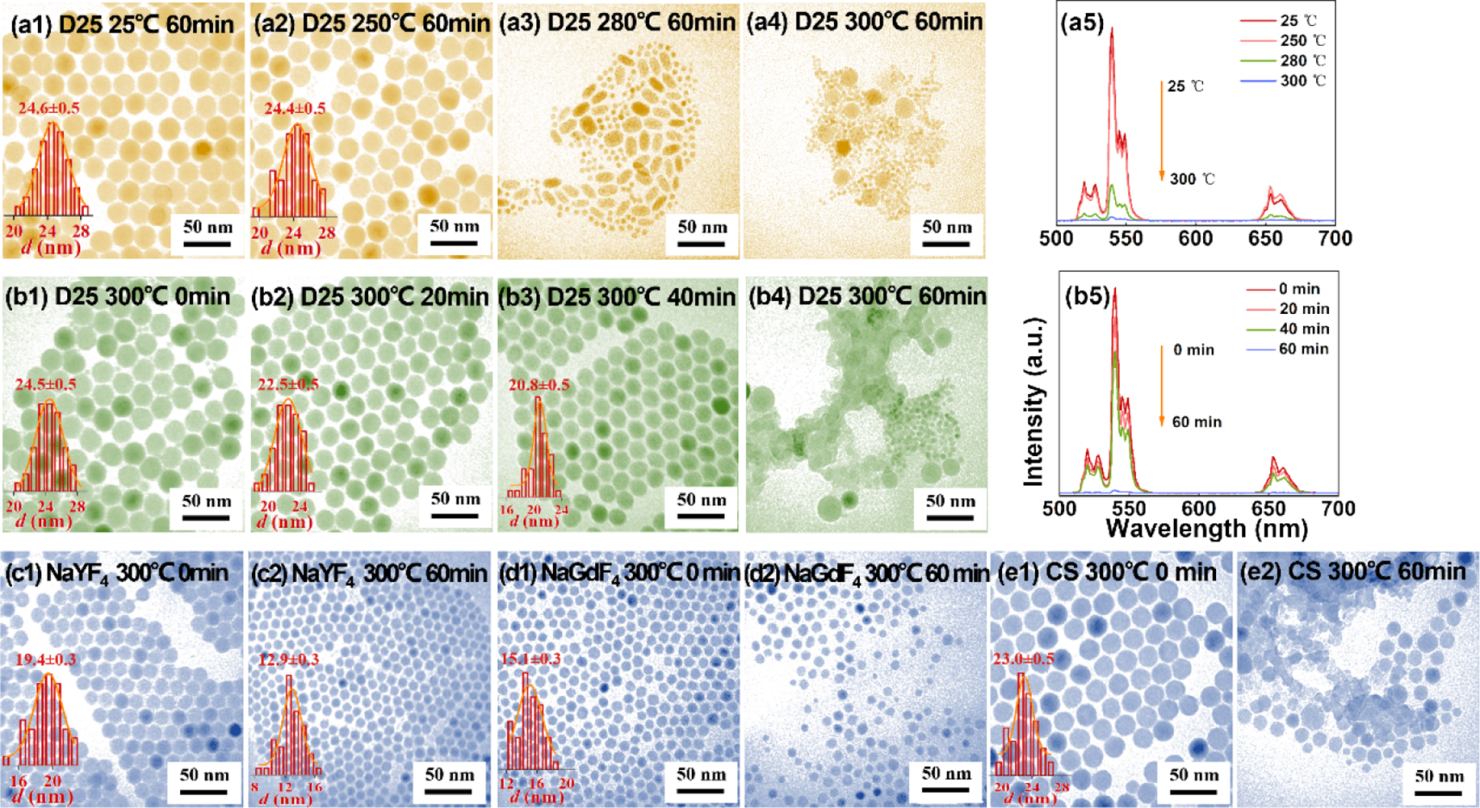
TEM images of different upconversion nanoparticles after post-annealing treatment at different temperatures in a mixture of OA (6 mL) and ODE (15 mL) for different times. (a1–a4) and (b1–b4) D25 nanoparticles (NaYF_4_: 20% Yb, 2% Er, ∼25 nm in diameter), (c1,c2) NaYF_4_ nanoparticles, (d1,d2) NaGdF_4_ nanoparticles, and (e1,e2) NaYF_4_: 20% Yb, 2% Er@NaLuF_4_ core–shell (CS) nanoparticles. Upconversion emission spectra of (a5) post-annealed D25 nanoparticles in a1–a4 and (b5) post-annealed D25 nanoparticles in panels (b1–b4) under continuous-wave (CW) 980 nm excitation (2 W cm^–2^). A histogram for particle size distribution was not added in panels (a3), (a4), (b4), (d2), and (e2) due to the breadth of the distribution. The used nanoparticle number density was ∼4.3 × 10^13^ mL^–1^ for panels (a1–a4) and (b1–b4).

Considering that temperature is a key parameter in nanoparticle synthesis, we first investigated its influence on the existing state of UCNPs in synthetic solvents, i.e., 1-octadecene (ODE) and oleic acid (OA). The stability of UCNPs was examined at elevated temperatures using a post-annealing approach. In a typical process, UCNPs were first synthesized using previously reported protocols^20,34^ and then annealed in a mixture of ODE and OA at different temperatures relevant to the nanoparticle synthesis conditions (see details in the SI).^35^ The amounts of the used nanoparticles were kept with the same equivalent total mole of rare earth ions (0.1 mmol) unless otherwise specified. The nanoparticle stability was assessed by morphological characterization using TEM and upconversion photoluminescent measurements (section 10 in the SI). A group of NaYF_4_: 20% Yb, 2% Er UCNPs with an average diameter of ∼25 nm in size (denoted as D25) was first studied. X-ray diffraction (XRD) characterization on these nanoparticles shows a typical hexagonal phase (Figure S1a). It was found that after post-annealing treatment in a mixture of 6 mL OA and 15 mL ODE (typical amounts for UCNP synthesis reaction) for 1 h, the nanoparticles were very stable at temperatures up to 250 °C with no apparent change in the size and shape (Figure 1a1,a2), associated with well-maintained UCL intensities (Figure 1a5). However, dramatic changes were found at further elevated temperatures (Figure 1a3,a4). When the post-annealing treatment temperature reached 280 °C, many small particles appeared in the sample with a much broader size distribution than the original ones and associated with irregular morphologies, some of which with a size down to several nanometers (Figure 1a3). When the post-annealing treatment temperature reached 300 °C, most resulting nanoparticles had a very small size of several nanometers, together with a small number of nanoparticles with larger sizes (also significantly smaller than the initial 25 nm) (Figure 1a4). These results present evidence for severe nanoparticle dissolution at the tested conditions, agreeing well with the speculation of Hudry et al. based on local chemical and structural analyses of core–shell UCNPs.^25^ The observed nanoparticle dissolution can explain the quenched UCL, particularly being almost completely quenched after the 300 °C treatment (Figure 1a5). Further investigation in a time sequence revealed that the nanoparticle dissolution is a slowly changing process (Figure 1b1–4), with a well-detectable change in the UCL property starting from 20 min (Figure 1b5).

The above studies were carried out on lanthanide-doped NaYF_4_ nanoparticles, embracing both skeleton rare-earth ions (Y^3+^) in the host matrix and optically active sensitizer (Yb^3+^) and activator (Er^3+^) ions. Due to the difference in the radius of rare-earth ions, the substitution of the skeleton cations with lanthanide dopants may induce distortions and defects in the crystal lattice, which may lead to more soluble doped nanoparticles. This consideration motivated us to investigate the influence of the composition of nanoparticles on their dissolution. For this purpose, undoped NaYF_4_ nanoparticles (∼19 nm) as well as NaGdF_4_ nanoparticles (∼15 nm) were synthesized, and both of them have a typical hexagonal phase confirmed by XRD characterization (Figure S1b). These nanoparticles were post-annealed 300 °C and changes in their size and morphology were then observed. As shown in Figure 1c2,d2, both undoped NaYF_4_ and NaGdF_4_ nanoparticles were found significantly dissolved, though to different degrees and with different features, being seemingly homogeneous for NaYF_4_ and inhomogeneous for NaGdF_4_.

The influence of the heterostructure of the nanoparticles on their dissolution was also studied. NaYF_4_: 20% Yb, 2% Er@NaLuF_4_ nanoparticles were synthesized using a regular shell epitaxial growth procedure (referring to section 5 in the SI). TEM characterization of the corresponding core and core–shell nanoparticles indicates an average thickness of ∼4 nm for the NaLuF_4_ shell (Figure S2, Figure 1e1). It was found that these core–shell nanoparticles were also subject to severe dissolution after the post-annealing treatment (Figure 1e2).

**Figure 2:**
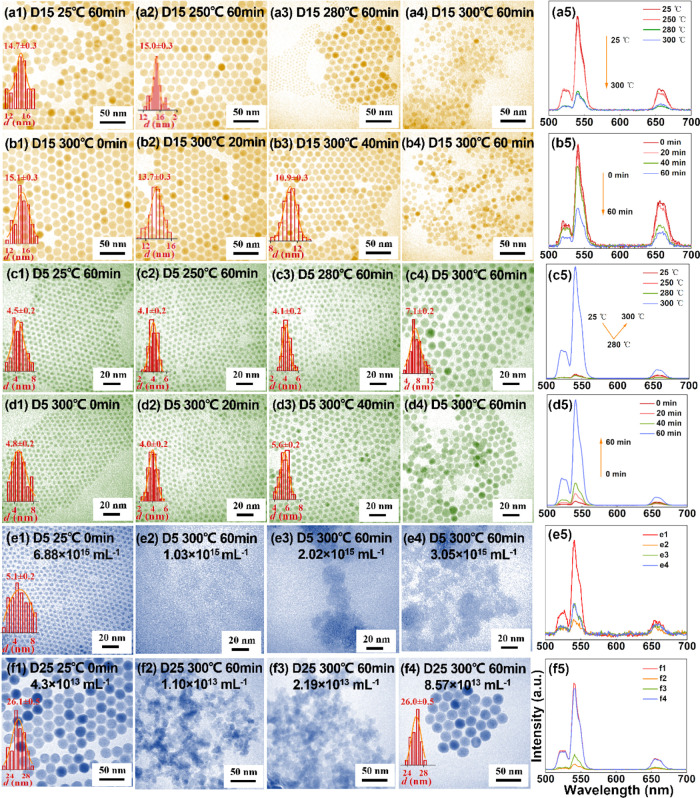
TEM images of different upconversion nanoparticles after post-annealing treatment at different temperatures in a mixture of OA (6 mL) and ODE (15 mL) for different times. (a1–a4, b1–b4) D15 nanoparticles (NaYF_4_: 20% Yb, 2% Er, ∼15 nm in diameter), (c1–c4, d1–d4) D5 nanoparticles (NaGdF_4_: 20% Yb, 2% Er, ∼5 nm in diameter). (e1–e4) D5 nanoparticles with different number densities. (f1–f4) D25 nanoparticles with different number densities. Upconversion emission spectra of (a5) post-annealed D15 nanoparticles in panels (a1–a4), (b5) D15 nanoparticles in panels (b1–b4), (c5) D5 nanoparticles in panels (c1–c4), (d5) D5 nanoparticles in panels (d1–d4), (e5) D5 nanoparticles in panels (e1–e4), and (f5) D25 nanoparticles in panels (f1–f4) under continuous-wave (CW) 980 nm excitation (4 W cm^–2^ for D5 nanoparticles, and 2 W cm^–2^ for D15 and D25 nanoparticles). The number density of D15 nanoparticles used is ∼2.02 × 10^14^ mL^–1^ in panels (a1–a4) and (b1–b4) and ∼6.88 × 10^15^ mL^–1^ in panels (c1–c4) and (d1–d4).

In the above studies, the size of the nanoparticles used was not constant, and the nanoparticle dissolution was well-identified for all these nanoparticles. However, we also realized that the variation in nanoparticle size may distract the observations. We therefore carried out more systematic studies on the size effect. Another two groups of NaYF_4_: 20% Yb, 2% Er and NaGdF_4_: 20% Yb, 2% Er nanoparticles were then synthesized, with average sizes of ∼15 nm (denoted as D15) and ∼5 nm (denoted as D5), respectively. The XRD characterization reveals that the D15 nanoparticles have a typical hexagonal phase (Figure S1c), while D5 nanoparticles are amorphous (Figure S1d). Their stability after the post-annealing treatment was examined. As shown in Figure 2, the D15 nanoparticles were also found significantly dissolved after the post-annealing treatment at 280 and 300 °C for 1 h (Figure 2a1–a4). In particular, the 300 °C post-annealing treatment led to many small nanoparticles with a much-reduced size down to 7–8 nm (Figure 2a4). Time sequential observations reveal a similar gradual nanoparticle dissolution (Figure 2b1–b4) as in the D25 sample. Accordingly, the UCL intensities were much quenched (Figure 2a5,b5).

Interestingly, in contrast to the observations for the D25 and D15 nanoparticles, we found that with the increase of the post-annealing temperature, the small size D5 nanoparticles gained size (Figure 2c1–c4), in line with significantly increased UCL intensities (Figure 2c5). Time sequential observations revealed more information regarding the gradual growth of these small nanoparticles at the tested conditions (Figure 2d1–d4), associated with increased UCL intensity (Figure 2d5).

The different phenomena for the D5 sample and the D15 and D25 groups prompted us to explore the underlying reason. We realized that although in the above experiments, the amount of the nanoparticles was kept the same in terms of the total mole of rare-earth ions (0.1 mmol), the particle number densities were quite different in the D5, D15, and D25 experiments because of their different sizes. The smaller the nanoparticle size, the larger was the particle number density in the experiments. By taking into account the average sizes extracted from the TEM images and the volume of the post-annealing solvents, the original number densities in the above D25, D15, and D5 experiments (Figure 1a1–a4,b1–b4, Figure 2a1–a4,b1–b4, Figure 2c1–c4,d1–d4) were estimated to be ∼4.3 × 10^13^, ∼2.02 × 10^14^, and ∼6.88 × 10^15^ mL^–1^, respectively, varying within almost two orders of magnitude^36^ (see calculation details in the SI).

We realized that the nanoparticle dissolution in the synthetic solvents at high temperatures may share similarities with the earlier identified nanoparticle disintegration in aqueous solutions, which is regulated by a dissolution equilibrium, with the nanoparticle number density as a key influential factor.^37,38^ The effect of particle number density on the stability of the UCNPs in the post-annealing treatment was then studied. More experiments with D5 and D25 nanoparticles were carried out with well-controlled nanoparticle number densities, with the morphologies of the resulting nanoparticles after post-annealing treatment at 300 °C for 1 h being characterized. As shown in Figure 2e1–e4 for the D5 nanoparticles, when the number density decreased to <3.05 × 10^15^ mL^–1^, a disastrous nanoparticle disintegration was observed, leading to severely quenched UCL (Figure 2e5). In the new investigations on the D25 nanoparticles, nanoparticle dissolution was always dramatic when the number density was lower than the previous value (∼2.19 × 10^13^ mL^–1^) (Figure 2f1–f3). However, when the number density was increased to ∼8.57 × 10^13^ mL^–1^, no significant changes in the nanoparticle size and morphology were found (Figure 2f4). It can also be seen from the corresponding UCL spectra (Figure 2f5) that the UCL intensity for the group with the highest nanoparticle number density was much better maintained than for the other groups, despite a noticeable intensity decrease compared to the original (indicating only slight structural change in the nanoparticles).

**Figure 3:**
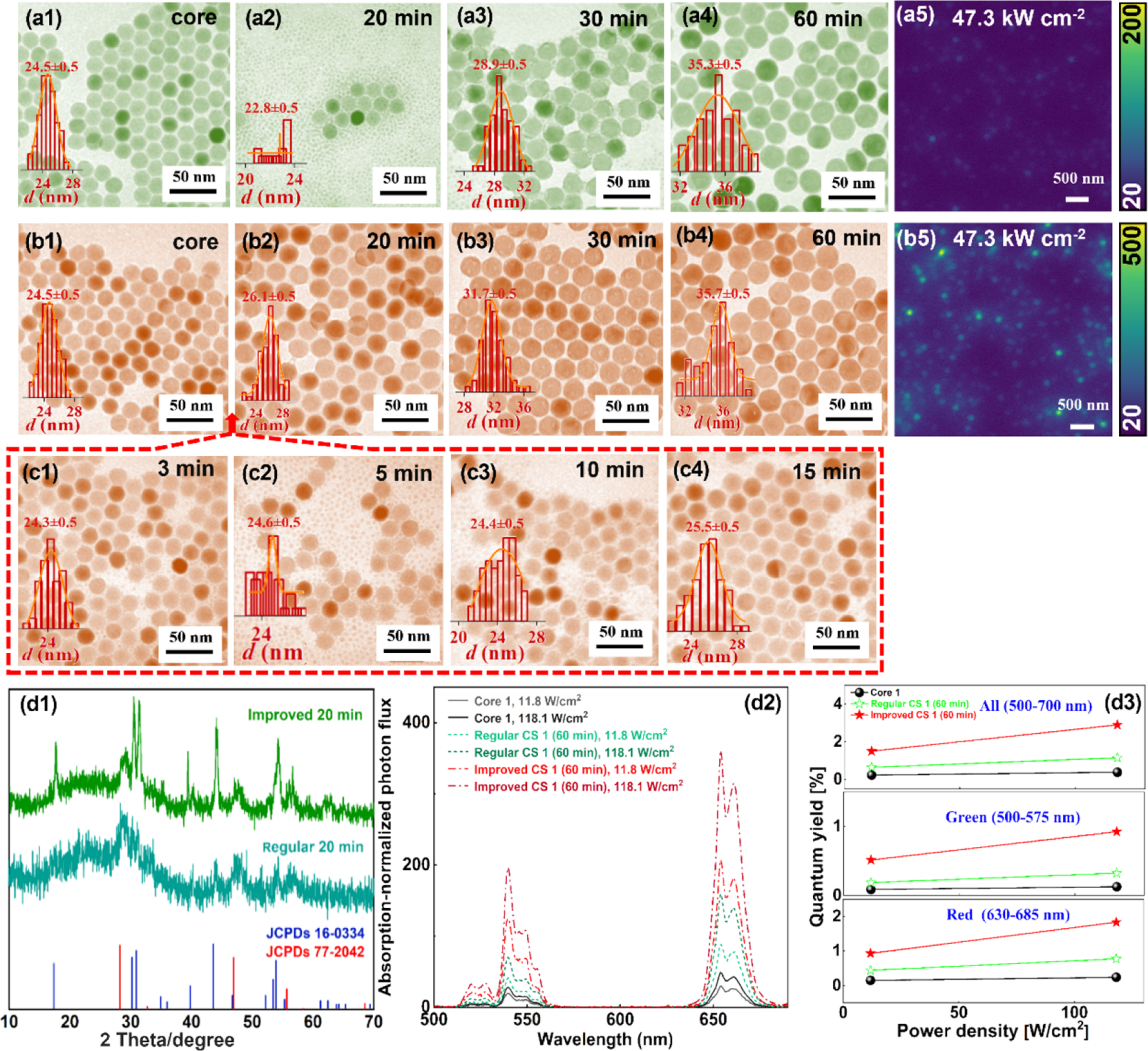
(a1–a4) TEM images of nanoparticles sampled at different times during the regular synthesis procedure of NaYF_4_: 20% Yb, 2% Er@NaLuF_4_ core–shell nanoparticles. (a5) Single particle imaging of nanoparticles in panel (a4) (CW 980 nm excitation, 47.3 kW cm^–2^). (b1–b4) TEM images of nanoparticles sampled at different times during the improved synthesis procedure of NaYF_4_: 20% Yb, 2% Er@NaLuF_4_ core–shell nanoparticles by adding excess sodium oleate. (b5) Single particle imaging of nanoparticles in panel (b4) (CW 980 nm excitation, 47.3 kW cm^–2^). Note that a1 and b1 are the same nanoparticle sample. (c1–c4) TEM images of nanoparticles sampled in 20 min during the improved synthesis procedure of NaYF_4_: 20% Yb, 2% Er@NaLuF_4_ core–shell nanoparticles by adding excess sodium oleate. (d1) XRD patterns of the nanoparticles in panels (a2) and (b2). (d2) Absorption-normalized upconversion emission spectra and (d3) upconversion quantum yields of nanoparticles in panels (a1)/(b1), (a4), and (b4) under CW 980 nm excitation of different power densities.

By means of these results, we have identified significant nanoparticle dissolution and reforming in the synthetic solvents at high temperatures, which is shown to be strongly dependent on the number density of the particles regardless of the nanoparticle size, composition, heterostructure, and phase. Interestingly, when re-examining previous reports, we found that UCNP dissolution after similar post-annealing treatments has been presented. For instance, in the report of Liu et al.,^35^ visible UCNP size changes can be noticed in the reported TEM images of the nanoparticles after post-annealing treatments above 280 °C (e.g., Figures 2 and 3a2 and Figure S5 in ref (35) but were overlooked by these authors.

In order to further figure out which solvent, OA or ODE, is decisive in dissolving the UCNPs, a control post-annealing experiment was performed using only ODE (21 mL) as the solvent. As seen in Figure S3, the D25 nanoparticles with the same number density were just slightly dissolved even after 60 min, in sharp contrast to the result in Figure 1b4. This reveals that oleic acid is dominantly etching the nanoparticles.

### History of Nanocrystal Dissolution and Growth in the Synthesis of Core–Shell UCNPs

The above experiments were conducted in high-temperature solvents, similar but not identical to the synthesis of core–shell nanoparticles. In the typical heating-up synthesis approach of core–shell nanoparticles, the core nanoparticles are usually added to the shell precursor solution at a lower temperature (significantly lower than 280 °C).^20^ We then carefully ran a heating-up synthesis procedure of core–shell UCNPs and tracked the change in size and morphology of the nanoparticles in the reaction mixture over time to monitor the growth process of the shell. In this experiment, the D25 nanoparticles were used as the seeds and a relatively low number density (∼2.02 × 10^14^ mL^–1^) was used. NaLuF_4_ was used as the shell material. It was found that the seed core nanoparticles were noticeably dissolved after 20 min of reaction at 300 °C with a reduced average diameter from ∼24.5 (Figure 3a1) to ∼22.8 nm (Figure 3a2). It was also noticed that there was a large number of tiny particles (several nanometers in diameter) at 20 min (Figure 3a2). XRD characterization of the nanoparticles sampled at 20 min shows that the cubic phase exists together with the hexagonal phase (Figure 3d1), indicating the production of cubic-phase nanocrystals formed by the shell precursors, in agreement with previous reports.^39−41^ Over time, the particles in the reaction mixture gradually grew into bigger but non-uniform ones with spherical-like shapes (Figure 3a3) and finally into large particles with uniform size and regular morphology at 60 min (Figure 3a4). The UCL intensity changes are also in line with the nanoparticle size changes described above, as shown in Figure 3d2. For instance, the overall absorption-normalized UCL photon flux (500–700 nm) increased by 2.8 folds for the so obtained final core–shell nanoparticles (sampled at 60 min) compared with that of the original core nanoparticles when the excitation power density is 11.8 W cm^–2^.

In view of the inhibition role of an increased number density on nanoparticle dissolution, we increased the number density of the core nanoparticles to ∼1.01 × 10^15^ mL^–1^ and repeated the core–shell synthesis. The core nanoparticles (Figure S4a) were then not found to be significantly dissolved at the same time point (20 min, Figure S4b) but that they directly grew to bigger and uniform core–shell nanoparticles (Figure S4c).

These results provide unequivocal evidence for a partial core dissolution process followed by an epitaxial growth process of the shell layer during the heating-up synthesis procedure of core–shell UCNPs. We have good reasons to believe that this is the major mechanism for the previously reported cation intermixing. It is relevant here to discuss other causes, e.g., cation diffusion and cation exchange. In a previous study on the thermal stability of core–shell UCNPs, Chen et al. proved that cation diffusion is sluggish at the standard core–shell synthesis temperature of 290 °C because of the insufficient vibrational energy of the atoms and a lack of vacant sites in the nanocrystals and that it may be well noticeable only above 350 °C.^23^ Compared to core dissolution followed by an epitaxial growth process of the shell layer involving substantial recrystallization and growth of the crystalline grains, it is also reasonable to speculate that the effect of cation exchange, though existing, is marginal. Our findings thus provide a simple but clear picture of the previously reported cation intermixing process in the interface of core–shell nanoparticles^25,35,42^ and can explain many phenomena reported in the literature. For example, Hudry et al. observed inter-diffusion of the shell element into the core giving rise to the formation of a nonhomogeneous solid solution characterized by concentration gradients and the lack of sharp interfaces,^25^ which is consistent with our observations. These authors also found that the thicker the shell layer, the smaller the left core portion (characterized by high-angle annular dark-field scanning transmission electron microscopy) of the synthetic core–shell particle becomes.^25^ This can also be well explained by the observed core nanoparticle partial dissolution process and its major limiting factor, i.e., the core nanoparticle number density. In the synthesis of core–shell nanoparticles, the shell thickness is generally controlled by changing the dose of core nanoparticles compared to the amount of the shell precursor. Usually, the thicker the shell layer is, the smaller the dose of the core nanoparticle can be used. In synthesizing thicker-shell core–shell nanoparticles, a smaller dose of core nanoparticles (in a certain amount of solvents) would experience more severe core nanoparticle dissolution, which would lead to a smaller left core portion. Our finding on the effect of core nanoparticle number density in the synthesis of core–shell nanoparticles can also explain the controversial observations in the literature regarding the existence of sharp core–shell interfaces.^21−25^ It can be speculated that dependent on the dose of the core nanoparticles in the synthesis of core–shell nanoparticles, both sharper interfaces and extended interfacial regions could occur due to different dissolution levels of the core nanoparticles.

### Suppression of Core Dissolution in the Synthesis of Core–Shell Upconversion Nanoparticles

We then sought means to suppress the core dissolution in the synthesis of core–shell UCNPs. We demonstrated that increasing the number density of the core nanoparticles may be a trivial but useful approach (Figure S4). However, this is not an ideal solution, as it would require a large amount of core nanoparticles. Especially, when the same batch of core nanoparticles is needed to synthesize a series of core–shell nanoparticles with different characteristics, the limited amount of core nanoparticles makes this approach infeasible (a typical synthesis involves the use of 1 mmol lanthanide ions^34^ and it is not trivial to scale up). We then sought other solutions.

We examined the similarity between the UCNP dissolution in the synthetic solvents and that in aqueous solutions.^37,38,43^ In aqueous solutions, it has been reported that the disintegration of nanoparticles is regulated by a dissolution equilibrium
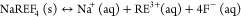
1where RE^3+^ represents rare earth ions, assuming that the ions are stoichiometrically dissolved.^37^ The equilibrium constant of NaREF_4_ nanoparticles, namely, the solubility product *K*_sp_, can be described by
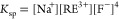
2

In the synthetic solvents (OA and ODE), a similar dissolution equilibrium for the involved ions should also exist. Lahtinen et al. found that the addition of F^–^ ions can effectively inhibit the dissolution of nanoparticles in an aqueous solution.^37^ Inspired by this, we then investigated whether the addition of F^–^ ions has the same inhibitory effect on the dissolution of core nanoparticles under the conditions of core–shell nanoparticle synthesis at high temperatures.

The effect of the addition of F^–^ ions on the inhibition of nanoparticle dissolution during the post-annealing treatment was first examined. Pre-synthesized D25 nanoparticles were used. It was found that after similar post-annealing treatment (300 °C, 1 h), the size and morphology of the used nanoparticles (with a relatively low nanoparticle number density ∼4.3 × 10^13^ mL^–1^) can be better preserved with increasing the dose of F^–^ ions, as shown in Figure S5, in stark contrast to the severe nanoparticle dissolution in the control experiment without the addition of F^–^ ions (Figure 1a4). These results show that the addition of F^–^ ions can indeed effectively inhibit the dissolution of nanoparticles in the synthetic solvents at high temperatures. However, it was later found that the addition of excessive F^–^ ions in the shell precursors can negatively affect the morphology of the resulting core–shell nanoparticles (Figure S6), in agreement with previous reports.^44,45^ It can be explained by the strong interaction between F^–^ and RE^3+^ ions to form insoluble compounds (REF_3_), which makes the growth of the shell layer less controllable. Therefore, it is concluded that the addition of excessive F^–^ ions in the core–shell nanoparticle synthesis is not a good solution to inhibit the dissolution of core nanoparticles.

We realized that Na^+^ ions may also have a role in regulating the dissolution equilibrium of the nanoparticles according to eq 2. To examine this conjecture, the same post-annealing treatment (300 °C for 1 h) as above was performed on the D25 nanoparticles (0.1 mmol) with addition of 0.5 mmol sodium oleate into the mixture of the solvents (6 mL OA, 15 mL ODE). TEM characterization reveals that though the size of the nanoparticles gradually decreased over time, the complete structure of the nanoparticles was still well retained after 60 min (Figure S7). This is in stark contrast to the result of nanoparticle destruction shown in Figure 1a4, b4, proving that addition of excess sodium oleate indeed has a protective effect on the structural integrity of the nanoparticles.

Due to the negligible interaction between Na^+^ and RE^3+^ ions, we anticipated that the addition of Na^+^ ions during the core–shell synthesis should have a less deleterious effect than F^–^ on the resulting core–shell nanoparticle morphology while inhibiting the core dissolution. Therefore, the core–shell synthesis process was adapted by introducing an excessive amount of Na^+^ ions by adding sodium oleate (NaOA) into the shell precursor (see experimental details in the SI). Other parameters remained the same as in previous experiments. Intriguingly, it was found that after adding 5 mmol of sodium oleate, no sign of seed (core) nanoparticle dissolution was observed at the same time points (20–30 min) in the core–shell synthesis procedure, and instead the seed nanoparticles already gained size (Figure 3b2,b3). It is also worthwhile noting that no tiny nanoparticle formation (by the shell precursors) was observed, in sharp contrast to the case of no addition of excessive amount of sodium oleate (Figure 3a2,a3). The absorption-normalized upconversion emission spectra of the core (Figure 3a1,b1), the regular (Figure 3a4), and the improved core–shell (Figure 3b4) nanoparticles, measured under CW 980 nm excitation of different power densities, are presented in Figure 3d2. As seen, the improved core–shell nanoparticles exhibit a remarkably stronger UCL intensity than the regular ones under the same excitation power density. We also compared the UCL intensity of the two groups of nanoparticles at a single particle level. As seen in Figure 3a5,b5, the improved group exhibits significantly higher UCL intensity than the reference group even under the high excitation intensity of 47.3 kW cm^–2^.

Note that in the above study on the effect of addition of excess sodium oleate on protecting nanoparticles, the nanoparticles still underwent a slight size decrease at 20 min even under the protection of sodium oleate (Figure S7). However, here in the adapted core–shell synthesis, the size of the core nanoparticles seemingly did not decrease at the early stage (<20 min) of the reaction. In order to figure out the details to further investigate the role of sodium oleate, this adapted core–shell synthesis was monitored in more detail by sampling more frequently at the early stage (3, 5, 10, 15 min) and performing TEM characterization on the products. As shown in Figure 3c1–c4, small sized nanoparticles were observed between 3 and 15 min, and their relative number density (compared to the seed core nanoparticles) reached the maximum at 5 min and then gradually decreased over time. At 20 min, almost all the small sized nanoparticles disappeared. XRD characterization carried out on the 20 min sampled nanoparticles of the improved group shows that both hexagonal and cubic phases exist in this sample, but with the former as the dominant one, see Figure 3d1. This is remarkably different from the result of the regular core–shell synthesis procedure, where the co-existence of the cubic phase is more prominent (Figure 3.d1). These results point at that the addition of excess sodium oleate can greatly facilitate the α → β phase transition in the reaction mixture, i.e., that consumption of the small sized α-phase nanoparticles and subsequent growth onto the β-phase seed core nanoparticles occurs, as is well supported by the literature.^39,40,46^ A control experiment with addition of excess OA (8 mL, the usual dosage is 6 mL), instead of NaOA, during the core–shell nanoparticle synthesis process reveals that OA does not have the same inhibition effect on the seed core nanoparticle dissolution and acceleration effect on the α → β phase transition in the reaction mixture (Figure S8).

The upconversion quantum yields of several key nanoparticle samples, including the core, the regular core–shell (60 min), and the improved core–shell (60 min), were then quantified, as shown in Figure 3d3. As seen, the quantum yield of the improved core–shell nanoparticles is significantly higher than that of the regular core–shell nanoparticles at the tested excitation power densities (11.8 and 118.1 W cm^–2^).

### Performance Boost of Core–Shell Upconversion Nanoparticles by Suppressing Cation Intermixing

**Figure 4:**
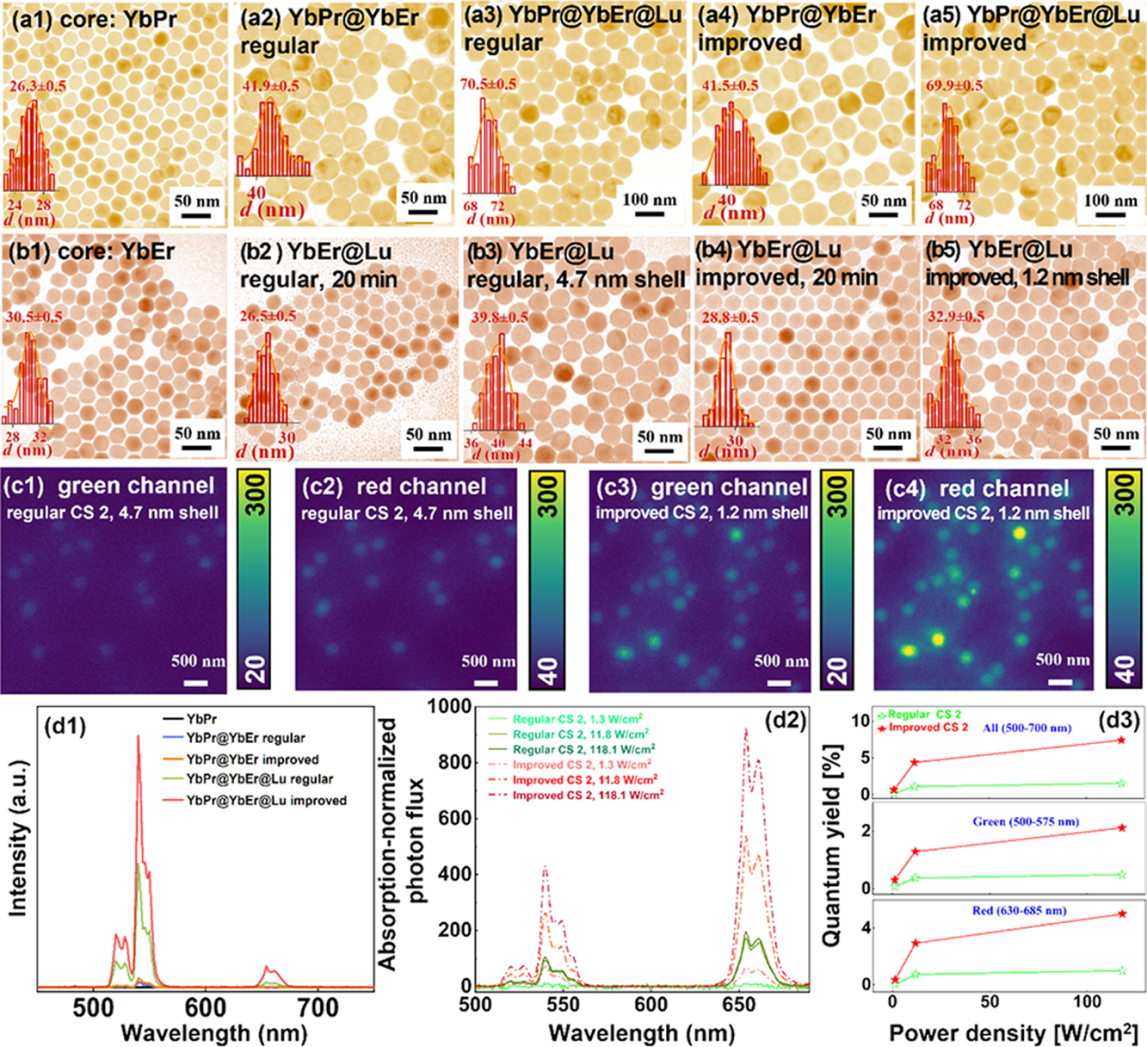
(a1) TEM image of core NaYF_4_: 15% Yb, 0.5% Pr nanoparticles. TEM images of (a2) core–shell NaYF_4_: 15% Yb, 0.5% Pr@NaYF_4_: 20% Yb, 2% Er nanoparticles and (a3) core–shell–shell NaYF_4_: 15% Yb, 0.5% Pr@NaYF_4_: 20% Yb, 2% Er@NaLuF_4_ nanoparticles synthesized by the regular synthesis method. TEM images of (a4) core–shell NaYF_4_: 15% Yb, 0.5% Pr@NaYF_4_: 20% Yb, 2% Er nanoparticles and (a5) core–shell–shell NaYF_4_: 15% Yb, 0.5% Pr@NaYF_4_: 20% Yb, 2% Er@NaLuF_4_ nanoparticles synthesized by the improved synthesis method. (b1) TEM image of core NaYF_4_: 20% Yb, 2% Er nanoparticles. TEM images of core–shell NaYF_4_: 20% Yb, 2% Er@NaLuF_4_ nanoparticles sampled at (b2) 20 min and (b3) 60 min synthesized by the regular core–shell synthesis method. TEM images of core–shell NaYF_4_: 20% Yb, 2% Er@NaLuF_4_ nanoparticles sampled at (b4) 20 min and (b5) 60 min synthesized by the improved core–shell synthesis method. Single particle imaging of nanoparticles in panel (b3) (excitation intensity is 47.3 kW cm^–2^): (c1) green channel; (c2) red channel. Single particle imaging of nanoparticles in panel (b5) (excitation intensity is 47.3 kW cm^–2^): (c3) green channel; (c4) red channel. (d1) Upconversion emission spectra of nanoparticles in panels (a1–a5) (CW 980 nm excitation, intensity 8 W cm^–2^). (d2) Absorption-normalized upconversion emission spectra of the nanoparticles in panels (b3) and (b5), measured under CW 980 nm excitation of different power densities. (d3) Upconversion quantum yields of nanoparticles in panels (b3) and (b5) under CW 980 nm excitation of different power densities.

The above results already clearly reveal the better UCL performance of the core–shell UCNPs when the cation intermixing is inhibited (Figure 3a5,b5,d2,d3). Further attempts were made to explore the utility of the proposed cation-intermixing inhibition approach. For this purpose, carefully designed core–shell−shell structured NaYF_4_: 15% Yb, 0.5% Pr@NaYF_4_: 20% Yb, 2% Er@NaLuF_4_ UCNPs were synthesized using the regular and the here improved core–shell synthesis procedure (see experimental details in the SI). This nanoparticle structure design was motivated by the consideration that a small amount of Pr^3+^ intermixed with Er^3+^ ions would spoil the UCL intensity of the latter. The outermost NaLuF_4_ shielding layer was included with the intention to protect the active YbPr@YbEr part from environmental quenching channels. This shielding layer was grown by our improved procedure in both experiments. The synthesized core nanoparticles and the regular and improved groups of the core–shell–shell UCNPs are presented in Figure 4a1–a5. As can be seen from the figure, the size of the core is ∼26.3 nm, the size of the core–shell is about ∼42 nm, and the thickness of the first shell is around 8 nm. Moreover, nanoparticle samples obtained by the two different methods showed similar average size and morphology. Further synthesis of core–shell–shell samples with a size of about 70 nm and with a thickness of the second layer of about 15 nm was accomplished. Similar to the core–shell sample, the core–shell–shell nanoparticles obtained by the two different methods have similar size and morphology. Interestingly, the UCL characterization shows that under the same excitation intensity, the improved group shows ∼2-fold UCL intensity as that of the regular group (Figure 4d1), confirming the better performance of the cation-intermixing-inhibited core–shell–shell nanoparticles.

We realized that the proposed cation-intermixing-inhibition procedure may reduce the requirement on the shell thickness to achieve a good protection for the emissive ions in the nanoparticles. To prove this speculation, we synthesized two groups of NaYF_4_: 20% Yb, 2% Er@NaLuF_4_ nanoparticles using the regular and improved core–shell synthesis procedures and compared their UCL properties. Here, in order to avoid the influence of different Na^+^ ion sources, sodium oleate was used as the Na^+^ source in the synthesis of core nanoparticles for both the regular and improved synthesis procedures. The pre-synthesized core nanoparticles have an average diameter of around 30.5 nm (Figure 4b1). The core–shell UCNPs synthesized using the improved procedure have an average diameter of ∼33 nm, revealing a shell thickness of ∼1.2 nm (Figure 4b5). TEM characterization on the nanoparticles sampled at 20 min confirmed that there was no severe core nanoparticle dissolution and that the α → β phase transition may have completed (Figure 4b4). For the regular group, the average diameter of the final nanoparticles is around 40 nm, indicating a nominally shell thickness of ∼4.7 nm (Figure 4b3). TEM characterization was performed on the nanoparticles sampled at 20 min, and a large number of small particles was found in the sample (Figure 4b2), indicating that the phase transition was not complete.

The absorption-normalized upconversion emission spectra of the regular (Figure 4b3) and the improved YbEr@Lu core–shell (Figure 4b5) nanoparticles, measured under CW 980 nm excitation of different power densities, are presented in Figure 4d2. As seen, the improved YbEr@Lu nanoparticles exhibit much stronger UCL brightness than the regular ones under the same excitation power density. The brightness of the two groups of nanoparticles were also compared at a single nanoparticle level. As seen in Figure 4c1–c4, the improved group shows significantly stronger UCL intensity for both the green and red channels under the same excitation intensity (47.3 kW cm^–2^). We further quantified the upconversion quantum yields of the regular (Figure 4b3) and the improved YbEr@Lu core–shell (Figure 4b5) nanoparticles, as shown in Figure 4d3. The quantum yield of the improved YbEr@Lu nanoparticles is significantly higher than that of the regular ones at the three tested excitation power densities (1.3, 11.8, and 118.1 W cm^–2^). At the power density of 118.1 W cm^–2^, the improved core–shell nanoparticles reach a surprisingly high quantum yield of 7.13%, which is around 4.8-fold of that of the regular ones (1.49%). The quantum yield values for different emission bands of the different samples are summarized in Table 1. Here, it is also worth noting that the quantum yield of the improved CS 2 group is significantly higher than that of the improved CS 1 group. This can be ascribed to the difference in the used Na^+^ source in the synthesis of the core nanoparticles, where sodium oleate was used for the improved CS 2 group and NaOH for the improved CS 1 group. It has been verified previously that avoidance of OH^–^ in the synthesis can greatly increase the optical performance of fluoride-based UCNPs.^14^ Taken together, the use of sodium oleate as the Na^+^ source in the core nanoparticle synthesis and the use of excess amount of the same chemical in the core–shell nanoparticle synthesis present the obtained impressively high quantum yield of core–shell UCNPs.

**Table 1:** Upconversion Quantum Yields of the Core, Regular, and Improved Core–Shell Nanoparticles

		Power density (W cm^–2^)		
Sample	Emission band	1.3	11.8	118.1
core 1	all (500–700 nm)		0.24%	0.36%
(dia. ∼24.5 nm)	green (500–575 nm)		0.09%	0.12%
	red (630–685 nm)		0.15%	0.24%
regular CS 1	all		0.63%	1.10%
(∼24.5 nm core, ∼5.4 nm shell)	green		0.19%	0.32%
	red		0.44%	0.78%
improved CS 1	all		1.44%	2.76%
(∼24.5 nm core, ∼5.6 nm shell)	green		0.51%	0.92%
	red		0.93%	1.84%
regular CS 2	all	0.12%	1.11%	1.49%
(∼30.5 nm core, ∼4.7 nm shell)	green	0.07%	0.37%	0.49%
	red	0.05%	0.74%	1.00%
improved CS 2	all	0.66%	4.24%	7.13%
(∼30.5 nm core, ∼1.2 nm shell)	green	0.31%	1.29%	2.12%
	red	0.35%	2.95%	5.01%

## Conclusions

In this work, the stability of lanthanide upconversion nanoparticles (UCNPs) in the synthetic solvents (octadecene and oleic acid) during post-annealing treatment was systematically investigated by transmission electron microscopy and spectroscopy. The results show that nanoparticle dissolution can occur when the reaction temperature is higher than 280 °C. Moreover, this phenomenon occurs regardless of nanoparticle size, composition, and structure. We confirmed that this instability of UCNPs imposes a harmful impact on the synthesis of core–shell nanoparticles when using the most widely used heating-up approach, leading to cation intermixing in the core and in the shell layer. Importantly, we established a simple method to better reserve the seed core nanoparticles from dissolution by adding excessive sodium oleate, which regulates the dissolution equilibrium of the core nanoparticles and facilitate the cubic-to-hexagonal phase transition involved in the core–shell synthesis process. This route can effectively suppress cation intermixing and present core–shell nanoparticles with a much-boosted performance. For instance, a surprisingly high quantum yield of 7.13% was obtained after ∼30.5 nm NaYF_4_: 20% Yb, 2% Er UCNPs were coated with an inert NaLuF_4_ shell of merely ∼1.2 nm in thickness. Our findings provide a clear explanation of the previously reported cation intermixing process in the interface of core–shell nanoparticles, i.e., partial or substantial core nanoparticle dissolution followed by epitaxial growth of the outer layer and ripening of the entire particle, and can explain many phenomena reported in the literature. We believe that the results of this work provide generic ramifications for synthesizing and employing core–shell UCNPs and further boost their applicability in already established technologies, including applications for bioimaging where a combination of small size and high brightness of the UCNPs is key for their applicability.

## Data Availability

All relevant raw data behind this study are available via DOI: 10.5281/zenodo.8017226.

## Supporting Information Available

Materials and reagents; synthesis details; nanomaterial characterization and multiphoton laser-scanning microscopy; calculation of number density of nanoparticles; supplementary figures (PDF)

## References

[ref1] Guo X.; Pu R.; Zhu Z.; Qiao S.; Liang Y.; Huang B.; Liu H.; Labrador-Páez L.; Kostiv U.; Zhao P.; Wu Q.; Widengren J.; Zhan Q. Achieving low-power single-wavelength-pair nanoscopy with NIR-II continuous-wave laser for multi-chromatic probes. Nat. Commun. 2022, 13, 2843 10.1038/s41467-022-30114-z. 35606360 PMC9126916

[ref2] Liu Z.; Qin X.; Liu X. Luminescence enrichment in perovskite-lanthanide composites: Complexity and complementarity. Handb. Phys. Chem. Rare Earths 2022, 61, 1–29. 10.1016/bs.hpcre.2021.12.002.

[ref3] Zhan Q.; Liu H.; Wang B.; Wu Q.; Pu R.; Zhou C.; Huang B.; Peng X.; Ågren H.; He S. Achieving high-efficiency emission depletion nanoscopy by employing cross relaxation in upconversion nanoparticles. Nat. Commun. 2017, 8, 1058 10.1038/s41467-017-01141-y. 29051497 PMC5648820

[ref4] Guo W.; Tan L.-L.; Li Q.; Li J.; Shang L. J. N. R. Upconversion nanorods anchored metal-organic frameworks via hierarchical and dynamic assembly for synergistic therapy. Nano Res. 2022, 15, 7533–7541. 10.1007/s12274-022-4324-4.

[ref5] Liu K.-C.; Zhang Z.-Y.; Shan C.-X.; Feng Z.-Q.; Li J.-S.; Song C.-L.; Bao Y.-N.; Qi X.-H.; Dong B. A flexible and superhydrophobic upconversion-luminescence membrane as an ultrasensitive fluorescence sensor for single droplet detection. Light: Sci. Appl. 2016, 5, e16136–e16136. 10.1038/lsa.2016.136. 30167183 PMC6059937

[ref6] Wang F.; Wen S.; He H.; Wang B.; Zhou Z.; Shimoni O.; Jin D. Microscopic inspection and tracking of single upconversion nanoparticles in living cells. Light: Sci. Appl. 2018, 7, 18007 10.1038/lsa.2018.7. 30839540 PMC5987356

[ref7] An R.; Liang Y.; Deng R.; Lei P.; Zhang H. Hollow nanoparticles synthesized via Ostwald ripening and their upconversion luminescence-mediated Boltzmann thermometry over a wide temperature range. Light: Sci. Appl. 2022, 11, 217 10.1038/s41377-022-00867-9. 35817780 PMC9273585

[ref8] Kotulska A. M.; Pilch-Wrobel A.; Lahtinen S.; Soukka T.; Bednarkiewicz A. Upconversion FRET quantitation: The role of donor photoexcitation mode and compositional architecture on the decay and intensity based responses. Light: Sci. Appl. 2022, 11, 256 10.1038/s41377-022-00946-x. 35986019 PMC9391450

[ref9] Huang G.; Liu Y.; Wang D.; Zhu Y.; Wen S.; Ruan J.; Jin D. Upconversion nanoparticles for super-resolution quantification of single small extracellular vesicles. eLight 2022, 2, 20 10.1186/s43593-022-00031-1. 35500253

[ref10] Ji Y.; Xu W.; Ding N.; Yang H.; Song H.; Liu Q.; Ågren H.; Widengren J.; Liu H. Huge upconversion luminescence enhancement by a cascade optical field modulation strategy facilitating selective multispectral narrow-band near-infrared photodetection. Light: Sci. Appl. 2020, 9, 184 10.1038/s41377-020-00418-0. 33298830 PMC7603315

[ref11] Han S.; Qin X.; An Z.; Zhu Y.; Liang L.; Han Y.; Huang W.; Liu X. Multicolour synthesis in lanthanide-doped nanocrystals through cation exchange in water. Nat. Commun. 2016, 7, 13059 10.1038/ncomms13059. 27698348 PMC5059460

[ref12] Peng T.; Pu R.; Wang B.; Zhu Z.; Liu K.; Wang F.; Wei W.; Liu H.; Zhan Q. The spectroscopic properties and microscopic imaging of thulium-doped upconversion nanoparticles excited at different NIR-II light. Biosensors 2021, 11, 148 10.3390/bios11050148. 34068452 PMC8151359

[ref13] Chen G.; Ågren H.; Ohulchanskyy T. Y.; Prasad P. N. Light upconverting core-shell nanostructures: Nanophotonic control for emerging applications. Chem. Soc. Rev. 2015, 44, 1680–1713. 10.1039/C4CS00170B. 25335878

[ref14] Homann C.; Krukewitt L.; Frenzel F.; Grauel B.; Würth C.; Resch-Genger U.; Haase M. NaYF_4_:Yb,Er/NaYF_4_ Core/Shell Nanocrystals with High Upconversion Luminescence Quantum Yield. Angew. Chem., Int. Ed. 2018, 57, 8765–8769. 10.1002/anie.201803083. 29732658

[ref15] Xie X.; Gao N.; Deng R.; Sun Q.; Xu Q.-H.; Liu X. Mechanistic Investigation of Photon Upconversion in Nd^3+^-Sensitized Core–Shell Nanoparticles. J. Am. Chem. Soc. 2013, 135, 12608–12611. 10.1021/ja4075002. 23947580

[ref16] Zhang Y.; Lei P.; Zhu X.; Zhang Y. Full shell coating or cation exchange enhances luminescence. Nat. Commun. 2021, 12, 6178 10.1038/s41467-021-26490-7. 34702817 PMC8548508

[ref17] Johnson N. J. J.; Korinek A.; Dong C.; van Veggel F. C. J. M. Self-Focusing by Ostwald Ripening: A Strategy for Layer-by-Layer Epitaxial Growth on Upconverting Nanocrystals. J. Am. Chem. Soc. 2012, 134, 11068–11071. 10.1021/ja302717u. 22734596

[ref18] Wang F.; Deng R.; Wang J.; Wang Q.; Han Y.; Zhu H.; Chen X.; Liu X. Tuning upconversion through energy migration in core-shell nanoparticles. Nat. Mater. 2011, 10, 968–973. 10.1038/nmat3149. 22019945

[ref19] Liang Y.; Zhu Z.; Qiao S.; Guo X.; Pu R.; Tang H.; Liu H.; Dong H.; Peng T.; Sun L. D.; Widengren J.; Zhan Q. Migrating photon avalanche in different emitters at the nanoscale enables 46th-order optical nonlinearity. Nat. Nanotechnol. 2022, 17, 524–530. 10.1038/s41565-022-01101-8. 35469009

[ref20] Qian H. S.; Zhang Y. Synthesis of hexagonal-phase core-shell NaYF_4_ nanocrystals with tunable upconversion fluorescence. Langmuir 2008, 24, 12123–12125. 10.1021/la802343f. 18839973

[ref21] Zhou B.; Tao L.; Chai Y.; Lau S. P.; Zhang Q.; Tsang Y. H. Constructing Interfacial Energy Transfer for Photon Up- and Down-Conversion from Lanthanides in a Core-Shell Nanostructure. Angew. Chem., Int. Ed. 2016, 55, 12356–12360. 10.1002/anie.201604682. 27377449

[ref22] Deng R.; Qin F.; Chen R.; Huang W.; Hong M.; Liu X. Temporal full-colour tuning through non-steady-state upconversion. Nat. Nanotechnol. 2015, 10, 237–242. 10.1038/nnano.2014.317. 25599189

[ref23] Chen B.; Peng D.; Chen X.; Qiao X.; Fan X.; Wang F. Establishing the Structural Integrity of Core-Shell Nanoparticles against Elemental Migration using Luminescent Lanthanide Probes. Angew. Chem., Int. Ed. 2015, 54, 12788–12790. 10.1002/anie.201506157. 26315850

[ref24] Li X.; Guo Z.; Zhao T.; Lu Y.; Zhou L.; Zhao D.; Zhang F. Filtration Shell Mediated Power Density Independent Orthogonal Excitations-Emissions Upconversion Luminescence. Angew. Chem., Int. Ed. 2016, 55, 2464–2469. 10.1002/anie.201510609. 26762564

[ref25] Hudry D.; Busko D.; Popescu R.; Gerthsen D.; Abeykoon A. M. M.; Kübel C.; Bergfeldt T.; Richards B. S. Direct Evidence of Significant Cation Intermixing in Upconverting Core@Shell Nanocrystals: Toward a New Crystallochemical Model. Chem. Mater. 2017, 29, 9238–9246. 10.1021/acs.chemmater.7b03118.

[ref26] Dong C.; van Veggel F. C. Cation exchange in lanthanide fluoride nanoparticles. ACS Nano 2009, 3, 123–130. 10.1021/nn8004747. 19206258

[ref27] Abel K. A.; Boyer J.-C.; Andrei C. M.; Van Veggel F. C. Analysis of the shell thickness distribution on NaYF_4_/NaGdF_4_ core/shell nanocrystals by EELS and EDS. J. Phys. Chem. Lett. 2011, 2, 185–189. 10.1021/jz101593g.

[ref28] van Veggel F. C. J. M.; Dong C.; Johnson N. J.; Pichaandi J. Ln^3+^–doped nanoparticles for upconversion and magnetic resonance imaging: Some critical notes on recent progress and some aspects to be considered. Nanoscale 2012, 4, 7309–7321. 10.1039/c2nr32124f. 23086529

[ref29] Bastian P. U.; Nacak S.; Roddatis V.; Kumke M. U. Tracking the motion of lanthanide ions within core–shell–shell NaYF_4_ nanocrystals via resonance energy transfer. J. Phys. Chem. C 2020, 124, 11229–11238. 10.1021/acs.jpcc.0c02588.

[ref30] Hudry D.; De Backer A.; Popescu R.; Busko D.; Howard I. A.; Bals S.; Zhang Y.; Pedrazo-Tardajos A.; Van Aert S.; Gerthsen D. Interface Pattern Engineering in Core-Shell Upconverting Nanocrystals: Shedding Light on Critical Parameters and Consequences for the Photoluminescence Properties. Small 2021, 17, 2104441 10.1002/smll.202104441. 34697908

[ref31] Bastian P. U.; Robel N.; Schmidt P.; Schrumpf T.; Günter C.; Roddatis V.; Kumke M. U. Resonance Energy Transfer to Track the Motion of Lanthanide Ions—What Drives the Intermixing in Core-Shell Upconverting Nanoparticles?. Biosensors 2021, 11, 515 10.3390/bios11120515. 34940272 PMC8699284

[ref32] Johnson N. J.; He S.; Diao S.; Chan E. M.; Dai H.; Almutairi A. Direct Evidence for Coupled Surface and Concentration Quenching Dynamics in Lanthanide-Doped Nanocrystals. J. Am. Chem. Soc. 2017, 139, 3275–3282. 10.1021/jacs.7b00223. 28169535

[ref33] Mhlongo G. H.; Dhlamini M. S.; Ntwaeaborwa O. M.; Swart H. C.; Hillie K. T. Luminescent properties and quenching effects of Pr^3+^ co-doping in SiO_2_:Tb^3+^/Eu^3+^ nanophosphors. Opt. Mater. 2014, 36, 732–739. 10.1016/j.optmat.2013.10.031.

[ref34] Li Z.; Zhang Y. An Efficient and User-Friendly Method for the Synthesis of Hexagonal-Phase NaYF_4_:Yb,Er/Tm Nanocrystals with Controllable Shape and Upconversion Fluorescence. Nanotechnology 2008, 19, 345606–345606. 10.1088/0957-4484/19/34/345606. 21730655

[ref35] Liu L.; Li X.; Fan Y.; Wang C.; El-Toni A. M.; Alhoshan M. S.; Zhao D.; Zhang F. Elemental Migration in Core/Shell Structured Lanthanide Doped Nanoparticles. Chem. Mater. 2019, 31, 5608–5615. 10.1021/acs.chemmater.9b01348.

[ref36] Hao S.; Shang Y.; Li D.; Agren H.; Yang C.; Chen G. Enhancing dye-sensitized solar cell efficiency through broadband near-infrared upconverting nanoparticles. Nanoscale 2017, 9, 6711–6715. 10.1039/C7NR01008G. 28485432

[ref37] Lahtinen S.; Lyytikäinen A.; Päkkilä H.; Hömppi E.; Perälä N.; Lastusaari M.; Soukka T. Disintegration of Hexagonal NaYF_4_:Yb^3+^, Er^3+^ Upconverting Nanoparticles in Aqueous Media: The Role of Fluoride in Solubility Equilibrium. J. Phys. Chem. C 2017, 121, 656–665. 10.1021/acs.jpcc.6b09301.

[ref38] Lisjak D.; Plohl O.; Ponikvar-Svet M.; Majaron B. Dissolution of upconverting fluoride nanoparticles in aqueous suspensions. RSC Adv. 2015, 5, 27393–27397. 10.1039/C5RA00902B.

[ref39] Rastogi C. K.; Lu E.; Tam J.; Pichaandi J. M.; Howe J.; Winnik M. A. Influence of the sodium precursor on the cubic-to-hexagonal phase transformation and controlled preparation of uniform NaNdF_4_ nanoparticles. Langmuir 2021, 37, 2146–2152. 10.1021/acs.langmuir.0c03346. 33534994

[ref40] Rinkel T.; Raj A. N.; Duhnen S.; Haase M. Synthesis of 10 nm beta-NaYF_4_:Yb,Er/NaYF_4_ Core/Shell Upconversion Nanocrystals with 5 nm Particle Cores. Angew. Chem., Int. Ed. 2016, 55, 1164–1167. 10.1002/anie.201508838. 26633748

[ref41] Wilhelm S.; Kaiser M.; Wurth C.; Heiland J.; Carrillo-Carrion C.; Muhr V.; Wolfbeis O. S.; Parak W. J.; Resch-Genger U.; Hirsch T. Water dispersible upconverting nanoparticles: effects of surface modification on their luminescence and colloidal stability. Nanoscale 2015, 7, 1403–1410. 10.1039/C4NR05954A. 25503253

[ref42] Dühnen S.; Haase M. Study on the Intermixing of Core and Shell in NaEuF_4_/NaGdF_4_ Core/Shell Nanocrystals. Chem. Mater. 2015, 27, 8375–8386. 10.1021/acs.chemmater.5b03846.

[ref43] Lisjak D.; Plohl O.; Vidmar J.; Majaron B.; Ponikvar-Svet M. Dissolution Mechanism of Upconverting AYF_4_:Yb,Tm (A = Na or K) Nanoparticles in Aqueous Media. Langmuir 2016, 32, 8222–8229. 10.1021/acs.langmuir.6b02675. 27459496

[ref44] Chen B.; Wang F. Recent advances in the synthesis and application of Yb-based fluoride upconversion nanoparticles. Inorg. Chem. Front. 2020, 7, 1067–1081. 10.1039/C9QI01358J.

[ref45] Li D.; Shao Q.; Dong Y.; Jiang J. Phase-, shape- and size-controlled synthesis of NaYF_4_:Yb^3+^,Er^3+^ nanoparticles using rare-earth acetate precursors. J. Rare Earths 2014, 32, 1032–1036. 10.1016/S1002-0721(14)60179-4.

[ref46] Xu L.; Wang M.; Chen Q.; Yang J.; Zheng W.; Lv G.; Quan Z.; Li C. Rare Earth Hydroxide as a Precursor for Controlled Fabrication of Uniform beta-NaYF_4_ Nanoparticles: A Novel, Low Cost, and Facile Method. Molecules 2019, 24, 357 10.3390/molecules24020357. 30669489 PMC6359501

