# Supplementary material for: Suppression of Cation Intermixing Highly Boosts the Performance of Core–Shell Lanthanide Upconversion Nanoparticles
Source: J Am Chem Soc. 2023 Aug 7;145(32):17621–31. doi: 10.1021/jacs.3c03019 (PMC10436270; doi:10.1021/jacs.3c03019)
Supplement: Supplementary file 1 — ja3c03019_si_001.pdf [file ja3c03019_si_001.pdf]

Supporting Information

Suppression of cation intermixing highly boosts the performance of core-shell lanthanide upconversion nanoparticles

Fuhua Huang[1,2,3], Niusha Bagheri[1], Li Wang [*2,3], Hans Ågren[*2,3], Jinglai Zhang[*2,3], Rui Pu[4], Qiuqiang Zhan[4,5], Yuhan Jing[6], Wen Xu[6], Jerker Widengren[1], Haichun Liu[*1]

[1] *Department of Applied Physics, KTH Royal Institute of Technology, S-10691, Stockholm, Sweden*
[2] *College of Chemistry and Molecular Sciences, Henan University, Kaifeng, Henan 475004, P. R. China*
[3] *Henan Center for Outstanding Overseas Scientists, Henan University, Kaifeng 475004, P. R. China*
[4] *Centre for Optical and Electromagnetic Research, Guangdong Provincial Key Laboratory of Optical Information Materials and Technology, South China Academy of Advanced Optoelectronics, South China Normal University, Guangzhou 510006, P.R. China*
[5] *MOE Key Laboratory of Laser Life Science, Guangdong Engineering Research Centre of Optoelectronic Intelligent Information Perception, South China Normal University, Guangzhou 510631, P.R. China*
[6] *Key Laboratory of New Energy and Rare Earth Resource Utilization of State Ethnic Affairs Commission, Key Laboratory of Photosensitive Materials & Devices of Liaoning Province, School of Physics and Materials Engineering, Dalian Minzu University, 18 Liaohe West Road, Dalian 11660, P.R. China*

1. Materials and Reagents

Yttrium (III) acetate hydrate (99.9%), erbium (III) acetate hydrate (99.9%), gadolinium (III) acetate hydrate (99.9%) were purchased from Sigma-Aldrich. Ytterbium (III) acetate hydrate (99.9%), lutetium (III) acetate hydrate (99.9%) were purchased from Adamas-beta. Sodium hydroxide (NaOH, >98%), ammonium fluoride (NH_4F, >99.99%), sodium oleate (NaOA, >97%), 1-octadecene (ODE, 90%), oleic acid (OA, 90%) were purchased from Aladdin®, China. Methanol (reagent grade),

*Corresponding authors.
E-mail addresses: haichun@kth.se (H. Liu), hans.agren@physics.uu.se (H. Ågren), chemwangl@henu.edu.cn (L. Wang), zhangjinglai@henu.edu.cn (J. Zhang)

ethanol (reagent grade), cyclohexane (reagent grade) were purchased from Sinopharm Chemical Reagent Co., China.

2. Synthesis of 25 nm $NaYF_4$:RE (RE= Yb, Er) nanocrystals

$NaYF_4$: 20% Yb, 2% Er nanocrystals were synthesized using a well-developed thermal decomposition method.[1] Typically, with the specific composition, a total amount of 1 mmol $RE(CH_3COO)_3$ was added to a mixture of oleic acid (6 mL) and 1-octadecene (15 mL) in a 250-mL flask at room temperature, and the mixture was heated at 160 °C and maintained for 30 min to form RE-oleate complexes. The resulting solution was cooled to room temperature, and then mixed with a methanol solution (10 mL) containing NH_4F (4 mmol) and NaOH (2.5 mmol). After that, the temperature of the mixture was increased to 120 °C and maintained for 10 min for a complete methanol removal. Then the solution was then degassed for 15 min to remove residual methanol and oxygen. Subsequently, the temperature of the resulting solution was quickly increased to 300 °C and maintained for 1 h in an argon atomsphere. The mixture cooled down to room temperature and the products were precipitated out with ethanol, collected by centrifugation at 10000 rpm for 10 min, washed with ethanol, and finally dispersed in 10 mL of cyclohexane.

3. Synthesis of 15 nm $NaYF_4$:RE (RE= Yb, Er) nanocrystals

$NaYF_4$: 20% Yb, 2% Er nanocrystals were synthesized using a well-developed thermal decomposition method.[1] Typically, with the specific composition, a total amount of 1 mmol $RE(CH_3COO)_3$ was added to a mixture of oleic acid (6 mL) and 1-octadecene (17 mL) in a 250-mL flask at room temperature, and the mixture was

heated at 160 °C and maintained for 30 min to form RE-oleate complexes. The resulting solution was cooled to room temperature, and then mixed with a methanol solution (10 mL) containing NH_4F (4 mmol) and NaOH (2.5 mmol). After that, the temperature of the mixture was increased to 120 °C and maintained for 10 min for a complete methanol removal. Then the solution was then degassed for 15 min to remove residual methanol and oxygen. Subsequently, the temperature of the resulting solution was quickly increased to 300 °C and maintained for 1 h in an argon atmosphere. The mixture cooled down to room temperature and the products were precipitated out with ethanol, collected by centrifugation at 10000 rpm for 10 min, washed with ethanol, and finally dispersed in 10 mL of cyclohexane.

4. Synthesis of 5 nm $NaGdF_4$:RE (RE= Yb, Er) nanocrystals

$NaGdF_4$: 20% Yb, 2% Er nanocrystals were synthesized using a well-developed thermal decomposition method.[1] Typically, with the specific composition, a total amount of 1 mmol $RE(CH_3COO)_3$ was added to a mixture of oleic acid (10 mL) and 1-octadecene (10 mL) in a 250-mL flask at room temperature, and the mixture was heated at 160 °C and maintained for 30 min to form RE-oleate complexes. The resulting solution was cooled to room temperature, and then mixed with a methanol solution (10 mL) containing NH_4F (4 mmol) and NaOH (2.5 mmol). After that, the temperature of the mixture was increased to 120 °C and maintained for 10 min for a complete methanol removal. Then the solution was degassed for 15 min to remove residual methanol and oxygen. Subsequently, the temperature of the resulting solution was quickly increased to 280 °C and maintained for 0.5 h in an argon atmosphere. The

mixture cooled down to room temperature and the products were precipitated out with ethanol, collected by centrifugation at 10000 rpm for 10 min, washed with ethanol, and finally dispersed in 10 mL of cyclohexane.

5. The regular synthesis method of core-shell structured nanocrystals

$NaYF_4$: 20% Yb, 2% Er core nanocrystals were first synthesized using the protocol described in section 3. $NaYF_4$: 20% Yb, 2% Er@$NaLuF_4$ nanoparticles were then synthesized using a well-developed method.[2] In a typical synthesis of $NaYF_4$: 20% Yb, 2% Er@$NaLuF_4$, 1 mmol $Lu(CH_3COO)_3$ was mixed with 6 mL oleic acid and 15 mL 1-octadecene in a 100 mL flask. The solution was heated to 150 °C to form a homogenous solution and then cooled down to room temperature. A suspension of the $NaYF_4$: 20% Yb, 2% Er core nanocrystals (0.5 mmol) dispersed in cyclohexane was added to the flask. The solution was maintained at 110 °C to remove the cyclohexane solvent and then subsequently cooled down to room temperature. A solution of 4 mmol NH_4F and 2.5 mmol NaOH in 10 mL of methanol was added to the flask and stirred for 30 min. Subsequently, the solution was heated to 100 °C to remove the methanol. After methanol was evaporated, the solution was heated to 300 °C and incubated at that temperature for 1 h under an argon atmosphere and then cooled to room temperature. The products were precipitated out with ethanol, collected by centrifugation at 10000 rpm for 10 min, washed with ethanol, and finally dispersed in cyclohexane.

6. The improved method of core-shell structured nanocrystals

$NaYF_4$: 20% Yb, 2% Er core nanocrystals were first synthesized using the protocol described in section 3. $NaYF_4$: 20% Yb, 2% Er@$NaLuF_4$ nanoparticles were then synthesized by the improved method in this paper. 1 mmol $Lu(CH_3COO)_3$ was mixed with 6 mL oleic acid and 15 mL 1-octadecene in a 100 mL flask, add 5 mmol of sodium oleate. The solution was heated to 150 °C to form a homogenous solution and then cooled down to room temperature. A suspension of the $NaYF_4$: 20% Yb, 2% Er core nanocrystals (0.5 mmol) dispersed in cyclohexane was added to the flask. The solution was maintained at 110 °C to remove the cyclohexane solvent and then subsequently cooled down to room temperature. A solution of 4 mmol NH_4F in 6 mL of methanol was added to the flask and stirred for 30 min. Subsequently, the solution was heated to 100 °C to remove the methanol. After methanol was evaporated, the solution was heated to 300 °C and incubated at that temperature for 1 h under an argon atmosphere and then cooled to room temperature. The products were precipitated out with ethanol, collected by centrifugation at 10000 rpm for 10 min, washed with ethanol, and finally dispersed in cyclohexane.

7. Synthesis of core $NaYF_4$: RE (RE= Yb, Pr or Yb, Er) nanocrystals

$NaYF_4$: 15% Yb, 0.5% Pr and $NaYF_4$: 20% Yb, 2% Er nanocrystals were synthesized using a well-developed thermal decomposition method.[1] Typically, with the specific composition, a total amount of 1 mmol $RE(CH_3COO)_3$ was added to a mixture of oleic acid (6 mL) and 1-octadecene (15 mL) in a 250-mL flask at room temperature, and the mixture was heated at 160 °C and maintained for 30 min to form RE-oleate complexes. The resulting solution was cooled to room temperature, and

then mixed with a methanol solution (10 mL) containing NH_4F (4 mmol) and NaOH (2.5 mmol). After that, the temperature of the mixture was increased to 120 °C and maintained for 10 min for a complete methanol removal. Then the solution was degassed for 15 min to remove residual methanol and oxygen. Subsequently, the temperature of the resulting solution was quickly increased to 300 °C and maintained for 1 h in an argon atomsphere. The mixture cooled down to room temperature and the products were precipitated out with ethanol, collected by centrifugation at 8000 rpm for 10 min, washed with ethanol, and finally dispersed in 10 mL of cyclohexane.

8. Synthesis of core-shell $NaYF_4$: 15% Yb, 0.5% Pr@$NaYF_4$: 20% Yb, 2% Er nanocrystals

In a typical synthesis of $NaYF_4$: 15% Yb, 0.5% Pr@$NaYF_4$: 20% Yb, 2% Er, 1 mmol $RE(CH_3COO)_3$ was mixed with 6 mL oleic acid and 15 mL 1-octadecene in a 100 mL flask. The solution was heated to 150 °C to form a homogenous solution and then cooled down to room temperature. A suspension of the $NaYF_4$: 15% Yb, 0.5% Pr core nanocrystals (0.5 mmol) dispersed in cyclohexane was added to the flask. The solution was maintained at 110 °C to remove the cyclohexane solvent and then subsequently cooled down to room temperature. A solution of 4 mmol NH_4F and 2.5 mmol NaOH in 10 mL of methanol was added to the flask and stirred for 30 min. Subsequently, the solution was heated to 100 °C to remove the methanol. After methanol was evaporated, the solution was heated to 300 °C and incubated at that temperature for 1 h under an argon atmosphere and then cooled to room temperature. The products were precipitated out with ethanol, collected by centrifugation at 10000

rpm for 10 min, washed with ethanol, and finally dispersed in cyclohexane. Please note that in the modified method presented here, the synthesis of this nanoparticle requires the addition of 5 mmol of sodium oleate during the synthesis of the precursor, while removing the NaOH added in the subsequent step. The other steps are the same as above.

9. Synthesis of core-shell-shell $NaYF_4$: 15% Yb, 0.5% Pr@$NaYF_4$: 20% Yb, 2% Er@$NaLuF_4$ nanocrystals

In a typical synthesis of $NaYF_4$: 15% Yb, 0.5% Pr@$NaYF_4$: 20% Yb, 2% Er@ $NaLuF_4$, 1 mmol $Lu(CH_3COO)_3$ was mixed with 6 mL oleic acid and 15 mL 1-octadecene in a 100 mL flask. The solution was heated to 150 °C to form a homogenous solution and then cooled down to room temperature. A suspension of the $NaYF_4$: 15% Yb, 0.5% Pr@$NaYF_4$: 20% Yb, 2% Er core-shell nanocrystals (0.5 mmol) dispersed in cyclohexane was added to the flask. The solution was maintained at 110 °C to remove the cyclohexane solvent and then subsequently cooled down to room temperature. A solution of 4 mmol NH_4F and 2.5 mmol NaOH in 10 mL of methanol was added to the flask and stirred for 30 min. Subsequently, the solution was heated to 100 °C to remove the methanol. After methanol was evaporated, the solution was heated to 300 °C and incubated at that temperature for 1 h under an argon atmosphere and then cooled to room temperature. The products were precipitated out with ethanol, collected by centrifugation at 10000 rpm for 10 min, washed with ethanol, and finally dispersed in cyclohexane. Please note that in the modified method presented here, the synthesis of these nanoparticles requires the

addition of 5 mmol of sodium oleate during the synthesis of the precursor, while removing the NaOH added in the subsequent step. The other steps are the same as above.

10. Characterization and multiphoton laser-scanning microscopy

Transmission electron microscopy (TEM) images were obtained on a JEOL JEM-2100 transmission electron microscope in the Analytical and Testing Center, School of Life Science, Henan University. Upconversion luminescence spectra in Fig. 1, Fig. 2, Fig. 4.d1 were recorded at room temperature with a HORIBA FluoroMax+ spectrofluorometer with a photomultiplier tube (R13456, Hamamatsu), equipped with a fiber-coupled diode laser at 980 nm (Changchun New Industry). Upconversion luminescence spectra and quantum yields in Fig. 3.d2-d3 and Fig. 4.d2-d3 were measured by a third-party agency, the Inspection and Test Center of Xiamen Rare-Earth Materials, Fujian Institute of Research on the Structure of Matter, China Academy of Sciences. The measurements were performed on an FLS980 spectrometer (Edinburgh Instruments) equipped with a photomultiplier tube (R928, Hamamatsu) and an integrating sphere. For quantum yield measurements, the measured photon flux, for both the excitation and the emission, was calibrated with a reference tungsten lamp.

11. Calculation of the number density of nanoparticles

D25: The volume of a single UCNP (ligand free) is $V_{UCNP}=4/3\times(d/2)^3\times\pi$ $=4/3\times(25/2)^3\times3.14$ $=8.18\times10^3$ nm^3, while the average mass of a single UCNP (ligand free) is $m_{UCNP}=\rho_{NaYF4}\times V_{UCNP}$ $= 4.23\times10^{-21}\times8.18\times10^3=3.46\times10^{-17}$ g. Considering the mass

loss of OA ligands, the average mass of initial OA-coated single UCNP is $m_{UCNP-OA}=3.46\times10^{-17}/80\%=4.33\times10^{-17}$ g. As a result, the “molecular weight” of initial OA-coated single UCNP is $M_{UCNP-OA}=m_{UCNP-OA}\times N_A= 4.33\times10^{-17}\times6.02\times10^{23}=2.60\times10^{7}$ g/mol. The total molar amount of 0.1 mmol nanoparticles is $0.026604/2.60\times10^{7}=1\times10^{-9}$ mol. At this time, the total number of particles in 0.1 mmol nanoparticles is $N_{UCNP-OA}=1\times10^{-9}\times6.02\times10^{23}=6.02\times10^{14}$. Finally, the number density of 0.1 mmol D25 nanoparticles in 14 mL solvent is $6.02\times10^{14}/14$ mL$=4.3\times10^{13}$ mL^{-1}.

D15: The volume of a single UCNP (ligand free) is $V_{UCNP}=4/3\times(d/2)^3\times\pi =4/3\times (15/2)^3\times3.14 =1.77\times10^3$ nm^3, while the average mass of a single UCNP (ligand free) is $m_{UCNP}= \rho_{NaYF4}\times V_{UCNP} = 4.23\times10^{-21}\times1.77\times10^3=7.47\times10^{-18}$ g. Considering the mass loss of OA ligands, the average mass of initial OA-coated single UCNP is $m_{UCNP-OA}=7.47\times10^{-18}/80\%=9.34\times10^{-18}$ g. As a result, the “molecular weight” of the initially OA-coated single UCNPs is $M_{UCNP-OA}=m_{UCNP-OA}\times N_A= 9.34\times10^{-18}\times6.02\times10^{23}=5.62\times10^{6}$ g/mol. The total molar amount of 0.1 mmol nanoparticles is $0.026604/5.62\times10^{6}=4.7\times10^{-9}$ mol. At this time, the total number of particles in 0.1 mmol nanoparticles is $N_{UCNP-OA}=4.7\times10^{-9}\times6.02\times10^{23}=2.83\times10^{15}$. Finally, the number density of 0.1 mmol D15 nanoparticles in 14 mL solvent is $2.83\times10^{15}/14$ mL$=2.02\times10^{14}$ mL^{-1}.

D5: The volume of a single UCNP (ligand free) is $V_{UCNP}=4/3\times(d/2)^3\times\pi =4/3\times (5/2)^3\times3.14 =65.42$ nm^3, while the average mass of a single UCNP (ligand free) is $m_{UCNP}= \rho_{NaYF4}\times V_{UCNP} = 4.23\times10^{-21}\times65.42=2.77\times10^{-19}$ g. Considering the mass loss of

OA ligands, the average mass of initial OA-coated single UCNP is $m_{UCNP\text{-}OA}=2.77\times10^{-19}/80\%=3.46\times10^{-19}$ g. As a result, the "molecular weight" of initial OA-coated single UCNP is $M_{UCNP\text{-}OA}=m_{UCNP\text{-}OA}\times N_A=$ $3.46\times10^{-19}\times6.02\times10^{23}=2.08\times10^{5}$ g/mol. The total molar amount of 0.1 mmol nanoparticles is $0.033439/2.08\times10^{5}=1.6\times10^{-7}$ mol. At this point, the total number of particles in 0.1 mmol nanoparticles is $N_{UCNP\text{-}OA}=1.6\times10^{-7}\times6.02\times10^{23}=9.63\times10^{16}$. Finally, the number density of 0.1 mmol D5 nanoparticles in 14 mL solvent is 9.63×10^{16}/14 mL=6.88×10^{15} mL^{-1}.

Supplementary Figures

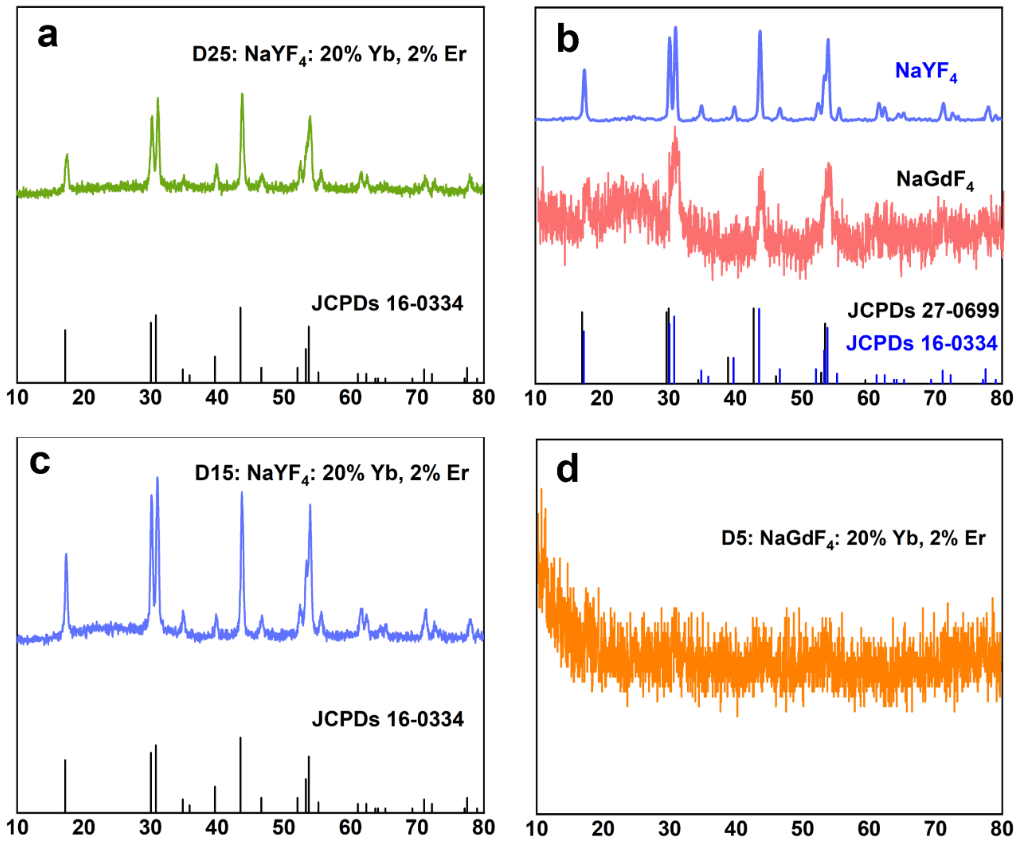

Fig. S1 XRD patterns of (a) D25 ($NaYF_4$: 20% Yb, 2% Er, 25 nm in diameter), (b) $NaGdF_4$ and $NaYF_4$ nanoparticles, (c) D15 ($NaYF_4$: 20% Yb, 2% Er, 15 nm in diameter), and (d) D5 ($NaGdF_4$: 20% Yb, 2% Er, 5 nm in diameter).

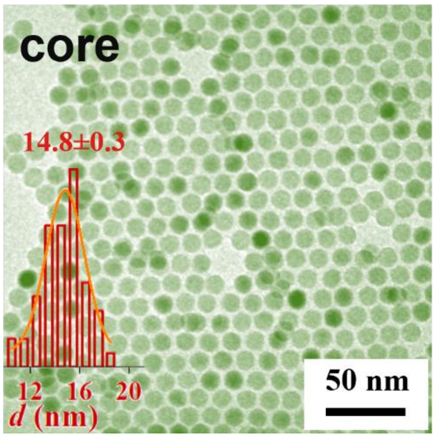

Fig. S2 TEM image of the used $NaYF_4$: 20% Yb, 2% Er seed core nanoparticles for the synthesis of core-shell nanoparticles in Fig. 1.e1

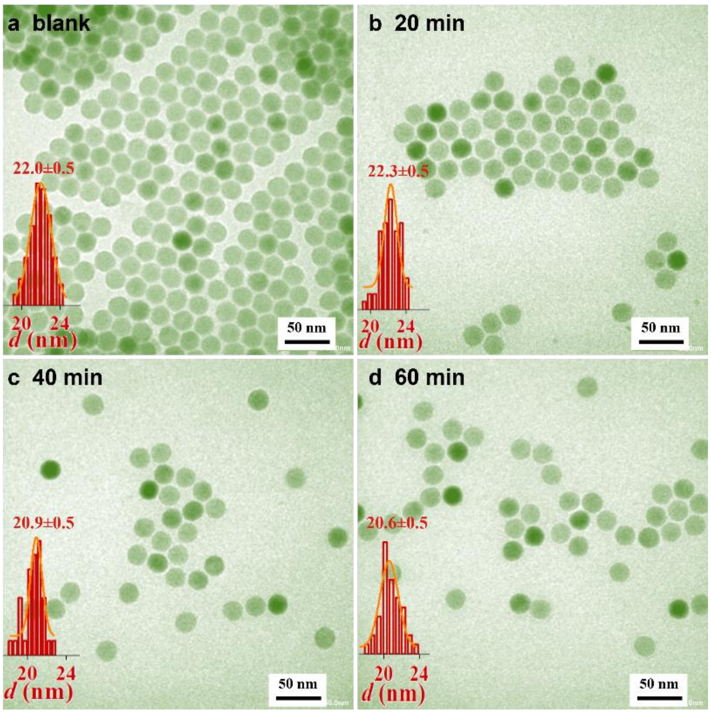

Fig. S3 TEM images of D25 upconversion nanoparticles after post-annealing treatment in ODE (21 mL) at 300 °C for different times. Nanoparticle number density: 4.3×10^{13} mL^{-1}.

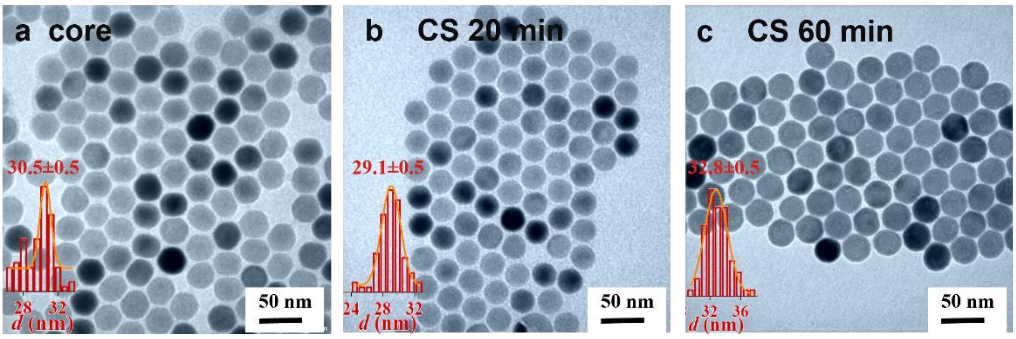

Fig. S4 TEM images of nanoparticles during a regular core-shell synthesis procedure sampled at different times. The number density of the core nanoparticles ($NaYF_4$: 20% Yb, 2% Er, 30 nm in diameter) is 1.01×10^{15} mL^{-1}.

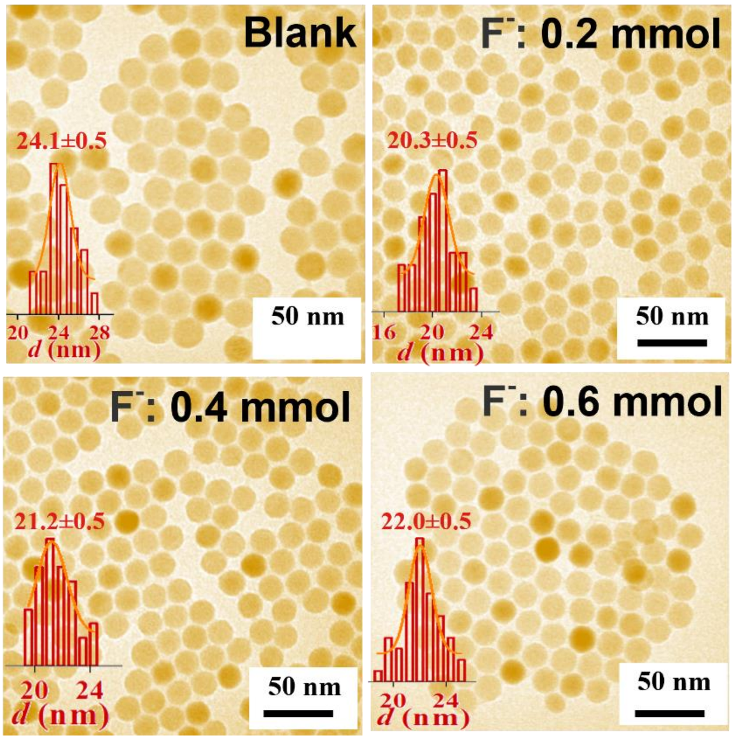

Fig. S5 TEM image of D25 nanoparticles affected by addition of F^- ion after the post-annealing treatment (300 °C, 1 h). The nanoparticle number density was 4.3×10^{13} mL^{-1}) for all the experiments.

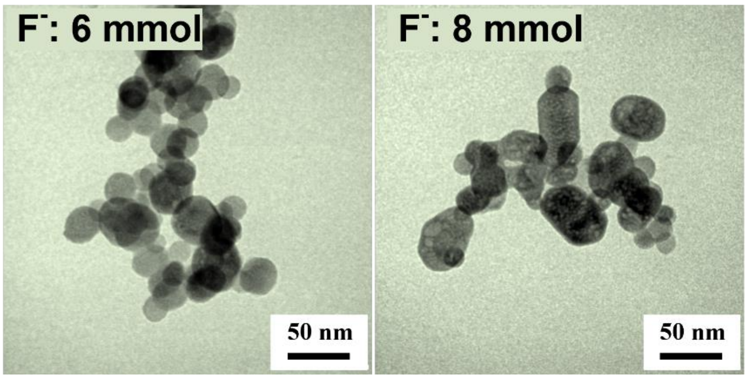

Fig. S6 TEM image of core-shell nanoparticles obtained by increasing the amount of F^- ion. The core nanoparticles are D25. The number density of the core nanoparticles is 4.3×10^{13} mL^{-1}.

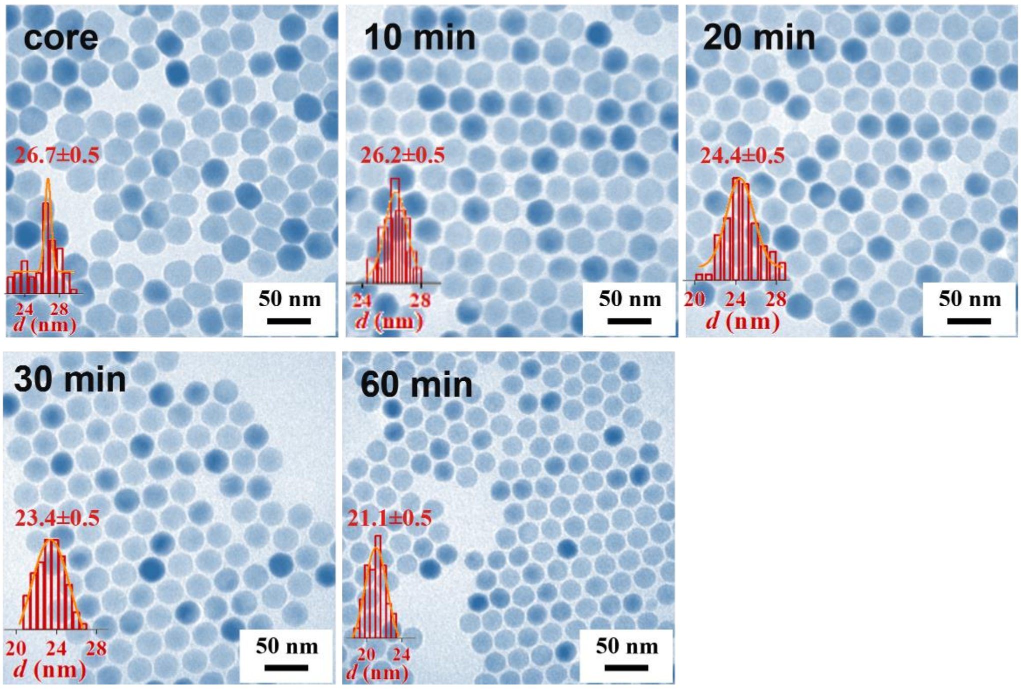

Fig. S7 TEM image of D25 nanoparticles affected by addition of NaOA after the post-annealing treatment (300 °C, 1 h). The nanoparticle number density was 4.3×10^{13} mL^{-1}) for all the experiments.

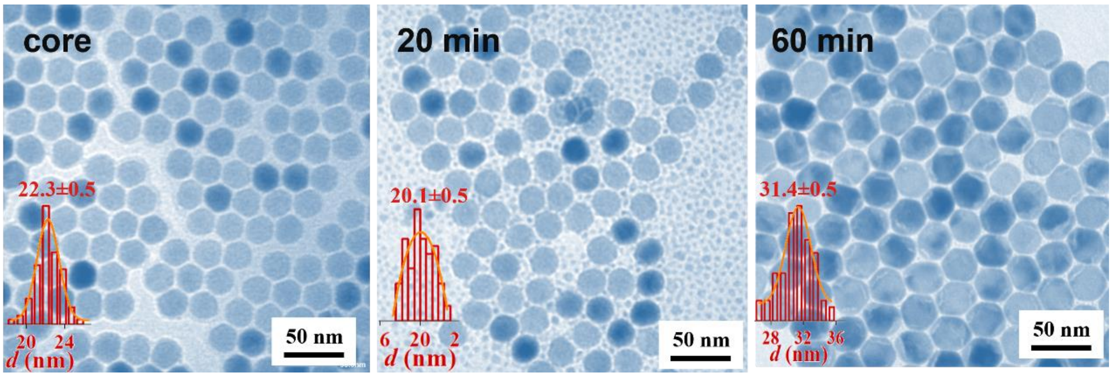

Fig. S8 TEM images of nanoparticles sampled at different times during an adapted core-shell synthesis procedure of $NaYF_4$: 20% Yb, 2% Er@$NaLuF_4$ nanoparticles by adding excess oleic acid.

References

1 Li, Z. & Zhang, Y. An Efficient and User-Friendly Method for the Synthesis of Hexagonal-Phase $NaYF_4$:Yb,Er/Tm Nanocrystals with Controllable Shape and

Upconversion Fluorescence. *Nanotechnology* **19**, 345606-345606 (2008).

2 Qian, H. S. & Zhang, Y. Synthesis of hexagonal-phase core-shell $NaYF_4$ nanocrystals with tunable upconversion fluorescence. *Langmuir* **24**, 12123-12125 (2008).
